# Education and Risk of Cancer in a Large Cohort of Men and Women in the United States

**DOI:** 10.1371/journal.pone.0003639

**Published:** 2008-11-04

**Authors:** Traci Mouw, Annemarie Koster, Margaret E. Wright, Madeleine M. Blank, Steven C. Moore, Albert Hollenbeck, Arthur Schatzkin

**Affiliations:** 1 Division of Epidemiology, Public Health & Primary Care, Faculty of Medicine, Imperial College London, London, United Kingdom; 2 Laboratory of Epidemiology, Demography, and Biometry, National Institute on Aging, National Institutes of Health, Bethesda, Maryland, United States of America; 3 Faculty of Health, Medicine and Life Sciences, Universiteit Maastricht, Maastricht, The Netherlands; 4 Department of Pathology, University of Illinois at Chicago, Chicago, Illinois, United States of America; 5 Division of Cancer Epidemiology and Genetics, National Cancer Institute, National Institutes of Health, Bethesda, Maryland, United States of America; 6 AARP, Washington, D. C., United States of America; University of Bern, Switzerland

## Abstract

**Background:**

Education inequalities in cancer incidence have long been noted. It is not clear, however, whether such inequalities persist in the United States, especially for less common malignancies and after adjustment for individual risk factors.

**Methodology/Principal Findings:**

Within the NIH–AARP Diet and Health Study, we examined the association between education and the risk of developing cancers in a prospective cohort of 498 455 participants who were 50–71 year old and without cancer at enrollment in 1995/96. During a maximum 8.2 years of follow–up we identified 40 443 cancers in men and 18 367 in women. In age-adjusted models, the least educated men (<high school), compared to those with the most education (post–graduate), had increased risks of developing cancers of the esophagus (RR: 2.64, 95%CI:1.86–3.75), head and neck (1.98, 1.54–2.54), stomach (2.32, 1.68–3.18), colon (1.31, 1.12–1. 53), rectum (1.68, 1.32–2.13), liver (1.90, 1.22–2.95), lung (3.67, 3.25–4.15), pleura (4.01, 1.91–8.42), bladder (1.56,1.33–1.83) and combined smoking–related cancers (2.41, 2.22–2.62). In contrast, lower education level was associated with a decreased risk of melanoma of the skin (0.43, 0.35–0.54) and local prostate cancers (0.79, 0.74–0.85). Women with the least education had increased risks of colon (1.60, 1.24–2.05), lung (2.14, 1.79–2.56), kidney (1.68, 1.12–2.54) and combined smoking–related cancers (1.66, 1.43–1.92) but a lower risk of melanoma of the skin (0.33, 0.22–0.51), endometrial (0.67, 0.51–0.89) and invasive breast cancers (0.72, 0.61–0.84). Adjustment for smoking and other risk factors did not eliminate these associations, except those for cancers of the head and neck, colon, and liver in men and kidney in women.

**Conclusions/Significance:**

We found a higher risk of malignant disease, particularly smoking– related cancers, among those in the lowest educational attainment category. Only some of the educational gradient is attributable to smoking. The persistence of substantial education inequalities in cancer incidence poses a challenge for etiologic research and public health policy.

## Introduction

Low socioeconomic status (SES) has been associated with increased risks of morbidity and mortality in different age groups within a variety of countries.[1] Education, an indicator of socioeconomic status, has been shown to be inversely associated with the incidence of cancer at several (but not all) anatomic sites.[2]–[6]–that is, in general, the higher the level of educational attainment, the lower the cancer risk.

A number of demographic, behavioral and biologic factors, including smoking, energy balance, cancer screening, hormone use and age at first birth, likely lie on the causal pathway between education and cancer.[7]–[9] Recent studies have shown that inflammation biomarkers, potentially causal with respect to cancer and overall mortality, are inversely associated with education.[10], [11] Multivariate adjustment for ‘unhealthy’ behaviors has been shown to completely eliminate the association between education and cancer incidence.[4] Although such analytic maneuvers may potentially explain the education–cancer connection, they do not obviate its public health importance.

Previous studies have investigated education in relation only to a single cancer or a few common malignancies. Only a few earlier studies in Europe have prospectively investigated multiple cancer sites, including relatively rare malignancies, with sufficient data to adjust for individual risk factors.[3], [4]. This study has two objectives: First, to determine whether educational inequalities for overall and site specific cancer incidence still exist in a large prospective US cohort; second, to investigate whether smoking and other lifestyle factors account for the observed (unadjusted) inequalities.

## Methods

### Study Population

The National Institutes of Health and AARP (formerly known as the American Association of Retired Persons) formed the NIH–AARP Diet and Health Study in 1995/96 when a 16–page paper questionnaire was mailed to 3.5 million AARP members aged 50–71 in 6 states (California, Florida, Louisiana, New Jersey, and Pennsylvania) and 2 metropolitan areas (Atlanta, Georgia and Detroit, Michigan). These states and metropolitan areas were selected because of the high quality of their cancer registries with a secondary goal of targeting areas with high minority populations. The cohort was designed to have a wide range of exposures in order to study the associations between health and lifestyle factors, especially diet. The study cohort and methods have been previously described in more detail.[12]

We obtained information on education, age, race, smoking, diet, alcohol consumption, weight, height, marital status, and personal and family history of cancer. Women answered an additional set of questions regarding their age at first birth, number of children, menopausal hormone use, and history of hysterectomy and oophorectomy. In addition, 334 643 participants reported their cancer screening behaviors on a second questionnaire mailed in 1996. A total of 566 402 participants provided sufficient information to be included in the cohort. Persons with prevalent cancers (n = 52 586), without information on education (n = 15 349), or who had moved or died before their questionnaire was received (n = 12) were excluded, leaving 498 455 participants (302 781 men, 195 674 women) for analysis.

### Procedures

We asked participants to report their highest grade or level of education completed in one of 7 categories: 8 yrs, 8–11 yrs, high school graduate, post high school training and technical college, some college, college graduate and post–graduate. Those who reported less than 8 years of education or 8–11 years of education were classified into a single category, less than high school. For each education category, we calculated age–adjusted incidence rates per 100,000 person-years by five year age intervals for individual cancer sites and all cancers in men and women separately. To examine whether smoking confounds the education-cancer associations, smoking status was included as a covariate in the age–adjusted models. Covariates entered into regression models included age (continuous), a 31-level smoking variable (combination of smoking status, time since quitting and smoking dose), race/ethnicity (White, Black, Hispanic, other), energy intake (continuous; Kcal/day), alcohol consumption (0, 0.1−<5, 5−<15, 15<30, 30+ grams/day), body mass index (BMI, (kg/m^2^); <25, 25−<30, 30−<35, 35+ kg/m^2^), physical activity (frequency of episodes that either lasted at least 20 minutes and increased breathing or heart rate, or led to working up a sweat: never/rarely, 1–3 time per month, 1–2 times per week, 3–4 times per week, 5+ times per week, unknown), marital status (yes/no), and family history of cancer (yes/no). For analyses of cancers of the breast, colon, ovary, and prostate, a variable for screening behavior during the three years prior to baseline (yes, no, missing) was included in the models. Information about menopausal hormone therapy (MHT) use (never, ever, missing) was included as a covariate in the analyses of cancers in women. For malignancies specific to women, we included a variable that combined a woman's age at first birth and number of children (no children, age at first birth <30 years with 1–2 children, age at first birth <30 years with 3+ children, and age at first birth ≥30 with any number of children). Each value of a categorical variable, including one for missing information, was included in the model as a separate variable, with the reference level excluded from the model.

We used probabilistic matching software to ascertain cancer endpoints through cancer registries in the original eight states and three additional states with the highest percentages of participants who had moved out of state during the follow-up period (Arizona, Nevada, and Texas). Participants were matched on their first and last names, sex, address histories, date of birth and Social Security Number (available for 85% of the participants). Address histories were constructed by annual linkage of cohort members to the National Change of Address database maintained by the U.S. Postal Service. We have shown that this method ascertains approximately 90% of incident cancers.[13]

Our end points were first primary incident cancers, defined according to the Surveillance Epidemiology and End Result (SEER) criteria, with minor modifications for malignancies of the head and neck, esophagus, pancreas, and prostate, as described previously.[14]–[18] Skin cancer was restricted to melanoma only. An *a priori* ‘smoking–related’ cancer was defined to comprise a malignancy of the head and neck, esophagus, lung, bladder, or pancreas.

The NIH–AARP Diet and Health Study protocol was approved by the U.S. National Cancer Institute Special Studies Institutional Review Board.

### Statistical methods

We used SAS software v 8.2 (Cary, NC) to calculate age–adjusted incidence rates. For cross tabulations and Cox proportional hazard regression models, we used Intercooled Stata 8.0 statistical software (College Station, TX). A participant's exit date was the time of the first of four possible events: 1) diagnosis with cancer; 2) a move outside the 11 states; 3) death; or 4) end of the study on December 31, 2003. We calculated relative risks (RR), equivalent to hazard ratios, and 95% confidence intervals (CI) from age– and multivariable–adjusted proportional hazards analyses, with time on study defined as the difference between the date of questionnaire return and the participant's exit date. We calculated tests for trend by including in Cox models a variable constructed from estimates of the number of years of education for each category of educational attainment. Specifically, we estimated 8 years of school for those with less than a high school education, 12 years for those who graduate high school, 13 years for post-high school trained individuals, 14 years for those who reported some college, 16 years for college graduates and 18 for post-graduate degree holders. We evaluated effect modification by stratified analysis and statistically with the use of a cross-product term. We present data only when at least 10 cancer cases occurred within an education category. All analyses were sex–stratified, with post–graduate education serving as the reference group. All tests were two sided and a p–value of less than or equal to 0.05 was considered statistically significant.

## Results

The relations between education and various risk factors are shown for men and women in Table 1. More educated men and women were more likely to be white, physically active, have a normal BMI, have never smoked, have been screened for cancer, consume fewer calories per day, drink more alcohol, and report a family history of cancer than less educated participants. More educated women were also more likely to have used MHT, to be nulliparous or, if parous, to have had their children later in life, than less educated women. More educated women were less likely to have had a hysterectomy but more likely to report intact ovaries.

**Table 1:** Baseline Characteristics According to Educational Attainment in Men and Women.

	Educational Attainment					
	Less than High School	Completed High School	Post High School Training	Some College	College Graduate	Post Graduate
Men						
No. of men	19 876	49 779	29 136	68 300	65 995	69 695
Median age, y	64.9	63.0	62.9	62.2	62.6	62.1
% White	88.8	93.4	93.6	92.2	93.3	93.5
%Current smoker	16.8	13.8	13.0	12.6	8.7	5.9
% Never smoker	17.6	24.2	23.8	23.8	32.6	42.7
Alcohol, % non–drinkers	9.7	7.4	5.7	6.0	4.7	4.8
Median total energy intake, kcal/d	1999.7	1942.4	1933.0	1871.5	1846.6	1830.8
BMI, % < = 25 kg/m^2^ a	23.9	24.6	26.8	26.3	31.6	34.8
% physically active 3–4/week	21.2	24.2	25.9	27.0	29.8	31.5
% married	84.8	86.1	86.3	84.8	86.2	85.7
% with family cancer history	44.9	45.6	46.6	46.9	47.5	48.2
% screening behavior b	41.8	48.4	53.0	53.9	58.4	61.3
Women						
No. of women	12 823	51 528	21 538	49 857	29 665	30 263
Median age, y	64.4	63.2	62.5	61.9	61.2	60.9
% White	82.0	91.3	90.8	89.8	88.9	90.1
% Current smoker	18.6	15.2	16.3	15.7	12.2	9.1
% Never smoker	40.5	47.2	43.1	40.4	45.4	48.7
Alcohol, % non–drinkers	18.1	11.4	9.8	8.9	7.8	7.7
Median total energy intake, kcal/d	1535.6	1471.3	1473.1	1443.1	1458.4	1441.6
BMI, % < = 25 kg/m^2^ a	30.4	38.9	40.6	43.7	49.4	49.5
% physically active 3–4/week	19.4	22.4	23.9	25.6	27.7	28.3
% married	42.5	47.5	45.7	42.8	45.7	41.7
% with family cancer history	50.5	51.0	52.1	50.7	51.2	51.6
% screening behavior b	44.9	54.1	57.4	59.2	62.5	65.4
% never had children	8.6	11.0	12.3	12.7	17.9	29.3
average age of first birth c	20.3	22.1	22.5	22.6	22.3	25.0
% hormone therapy, ever	39.8	45.0	53.1	57.3	58.1	60.4
% who had a hysterectomy	47.3	42.4	44.6	42.6	36.4	32.8
% who still have both ovaries	64.5	68.7	67.9	69.4	73.2	74.4

a BMI, body mass index (calculated as weight in kilograms divided by height in meters squared).

b Reported screening 3 years prior to baseline for colon, breast (women only), ovarian (women only) or prostate (men only) cancers.

c Among parous women.

### Age-adjusted models

The average follow–up time for the entire cohort was 6.86 years for a total contribution of 3 418 703 person years. In age–adjusted models, we found a significantly increased risk of any cancer for men with less than high school compared to men with post–graduate education (RR = 1.15, 95% CI = 1.10–1.19) (Table 2). The association was stronger for the subset of smoking–related cancers combined, with age–adjusted relative risks of 2.41 (2.22–2.62) in men who had less than high school education compared to men with post–graduate education. Men with less than high school, compared to those with post–graduate, education had significantly increased risks of developing cancers of the esophagus (2.64, 1.86–3.75), head and neck (1.98,1.54–2.54), stomach (2.32, 1.68–3.18), colon (1.31, 1.12–1. 53), rectum (1.68, 1.32–2.13), liver (1.90, 1.22–2.95), pleura (4.01, 1.91–8.42) and bladder (1.56,1.33–1.83) (Table 2). In contrast, men with less than a high school education had significantly decreased risks of localized prostate cancer (0.79, 0.74–0.85), as well as melanoma of the skin (0.43, 0.35–0.54) (Table 2).

**Table 2:** Relative Risk (RR) of Total Incident Cancer and of Site-Specific Cancer by Educational Attainment in Men.

Educational Attainment								p for trend a
		Less than High School	Completed High School	Post High School Training	Some College	College Graduate	Post Graduate	
All Cancer								
	Cases, No.	3085	6938	3920	9000	8961	8539	
	Age Adjusted Rate b	2156.27	2032.48	1976.83	1981.52	2004.85	1835.25	
	Age Adjusted RR (95% CI)	1.15(1.10,1.19)	1.11(1.07,1.14)	1.07(1.03,1.11)	1.08(1.05,1.11)	1.09(1.06,1.12)	1.00	<0.001
	Age, Smoking Adjusted RR(95%CI)	1.04(1.00,1.09)	1.03(1.00,1.07)	1.01(0.97,1.05)	1.01(0.98,1.04)	1.05(1.02,1.08)	1.00	0.249
	Multivariate Adjusted RR (95% CI) c	1.03(0.99,1.07)	1.02(0.99,1.06)	1.00(0.96,1.04)	1.00(0.97,1.03)	1.05(1.02,1.08)	1.00	0.648
Smoking Related Cancer d								
	Cases, No.	1016	1838	957	2232	1790	1314	
	Age Adjusted Rate b	722.11	539.11	481.99	492.53	400.38	284.16	
	Age Adjusted RR (95% CI)	2.41(2.22,2.62)	1.90(1.77,2.04)	1.69(1.56,1.84)	1.74(1.62,1.86)	1.41(1.31,1.51)	1.00	<0.001
	Age, Smoking Adjusted RR(95%CI)	1.58(1.45,1.72)	1.38(1.29,1.48)	1.26(1.16,1.37)	1.27(1.19,1.36)	1.19(1.11,1.28)	1.00	<0.001
	Multivariate Adjusted RR (95% CI)	1.54(1.42,1.68)	1.35(1.26,1.46)	1.24(1.14,1.35)	1.26(1.18,1.35)	1.19(1.11,1.28)	1.00	<0.001
Esophageal Cancer								
	Cases, No.	58	88	47	134	94	70	
	Age Adjusted Rate b	41.51	25.90	23.61	29.39	21.01	15.09	
	Age Adjusted RR (95% CI)	2.64(1.86,3.75)	1.71(1.25,2.35)	1.57(1.08,2.27)	1.96(1.47,2.62)	1.39(1.02,1.90)	1.00	<0.001
	Age, Smoking Adjusted RR(95%CI)	2.07(1.45,2.94)	1.42(1.03,1.95)	1.30(0.90,1.89)	1.62(1.21,2.16)	1.25(0.92,1.71)	1.00	<0.001
	Multivariate Adjusted RR (95% CI)	2.00(1.39,2.86)	1.38(1.00,1.89)	1.27(0.87,1.84)	1.59(1.18,2.13)	1.24(0.91,1.69)	1.00	<0.001
Head and Neck Cancer								
	Cases, No.	98	185	98	227	194	167	
	Age Adjusted Rate b	74.68	54.92	49.51	49.62	43.39	35.56	
	Age Adjusted RR (95% CI)	1.98(1.54,2.54)	1.54(1.25,1.89)	1.39(1.08,1.79)	1.40(1.14,1.70)	1.22(0.99,1.50)	1.00	<0.001
	Age, Smoking Adjusted RR(95%CI)	1.32(1.02,1.70)	1.14(0.93,1.41)	1.07(0.83,1.38)	1.06(0.87,1.30)	1.06(0.86,1.31)	1.00	0.033
	Multivariate Adjusted RR (95% CI)	1.29(0.99,1.67)	1.13(0.91,1.40)	1.07(0.83,1.37)	1.07(0.87,1.31)	1.07(0.87,1.32)	1.00	0.061
Stomach Cancer								
	Cases, No.	66	91	43	134	99	91	
	Age Adjusted Rate b	45.34	26.90	21.64	29.44	22.11	19.58	
	Age Adjusted RR (95% CI)	2.32(1.68,3.18)	1.36(1.02,1.83)	1.11(0.77,1.59)	1.51(1.16,1.97)	1.13(0.85,1.50)	1.00	<0.001
	Age, Smoking Adjusted RR(95%CI)	1.90(1.37,2.62)	1.18(0.88,1.58)	0.96(0.67,1.39)	1.31(1.00,1.71)	1.05(0.79,1.39)	1.00	<0.001
	Multivariate Adjusted RR (95% CI)	1.67(1.20,2.33)	1.11(0.82,1.49)	0.93(0.64,1.34)	1.25(0.95,1.63)	1.03(0.78,1.37)	1.00	0.007
Colon Cancer e								
	Cases, No.	228	533	324	580	579	547	
	Age Adjusted Rate b	160.92	156.08	163.19	127.55	129.53	117.52	
	Age Adjusted RR (95% CI)	1.31(1.12,1.53)	1.32(1.18,1.49)	1.38(1.20,1.58)	1.09(0.97,1.22)	1.10(0.98,1.23)	1.00	<0.001
	Age, Smoking Adjusted RR(95%CI)	1.22(1.04,1.43)	1.25(1.11,1.41)	1.31(1.14,1.51)	1.02(0.91,1.15)	1.06(0.94,1.19)	1.00	<0.001
	Multivariate Adjusted RR (95% CI)	1.10(0.94,1.29)	1.16(1.03,1.31)	1.25(1.09,1.43)	0.97(0.87,1.10)	1.04(0.93,1.17)	1.00	0.023
Rectum Cancer								
	Cases, No.	103	246	134	261	193	198	
	Age Adjusted Rate b	72.27	72.32	67.53	57.39	43.20	42.48	
	Age Adjusted RR (95% CI)	1.68(1.32,2.13)	1.70(1.41,2.05)	1.59(1.27,1.98)	1.35(1.12,1.62)	1.01(0.83,1.24)	1.00	<0.001
	Age, Smoking Adjusted RR(95%CI)	1.53(1.20,1.95)	1.59(1.32,1.93)	1.49(1.20,1.86)	1.26(1.05,1.52)	0.98(0.80,1.19)	1.00	<0.001
	Multivariate Adjusted RR (95% CI)	1.50(1.17,1.92)	1.56(1.29,1.89)	1.47(1.18,1.84)	1.25(1.04,1.51)	0.97(0.79,1.18)	1.00	<0.001
Liver Cancer								
	Cases, No.	32	61	30	76	75	54	
	Age Adjusted Rate b	23.05	17.75	15.10	16.67	16.77	11.60	
	Age Adjusted RR (95% CI)	1.90(1.22,2.95)	1.54(1.07,2.23)	1.30(0.83,2.03)	1.44(1.02,2.05)	1.44(1.02,2.05)	1.00	0.006
	Age, Smoking Adjusted RR(95%CI)	1.66(1.07,2.59)	1.39(0.96,2.02)	1.18(0.75,1.85)	1.30(0.91,1.85)	1.37(0.96,1.94)	1.00	0.045
	Multivariate Adjusted RR (95% CI)	1.28(0.82,2.01)	1.22(0.84,1.77)	1.07(0.68,1.67)	1.17(0.82,1.67)	1.31(0.93,1.87)	1.00	0.462
Pancreatic Cancer								
	Cases, No.	63	125	68	173	157	149	
	Age Adjusted Rate b	45.48	36.63	34.06	38.23	35.09	32.18	
	Age Adjusted RR (95% CI)	1.30(0.97,1.75)	1.13(0.89,1.44)	1.06(0.79,1.41)	1.19(0.95,1.48)	1.09(0.87,1.36)	1.00	0.085
	Age, Smoking Adjusted RR(95%CI)	1.15(0.85,1.55)	1.04(0.82,1.32)	0.98(0.73,1.31)	1.10(0.88,1.37)	1.05(0.84,1.31)	1.00	0.478
	Multivariate Adjusted RR (95% CI)	1.13(0.83,1.53)	1.02(0.80,1.30)	0.97(0.72,1.29)	1.09(0.87,1.36)	1.04(0.83,1.30)	1.00	0.581
Lung Cancer								
	Cases, No.	567	964	503	1092	807	479	
	Age Adjusted Rate b	401.23	282.69	253.66	241.17	180.56	104.09	
	Age Adjusted RR (95% CI)	3.67(3.25,4.15)	2.72(2.44,3.04)	2.44(2.15,2.77)	2.33(2.10,2.60)	1.74(1.55,1.95)	1.00	<0.001
	Age, Smoking Adjusted RR(95%CI)	2.02(1.79,2.29)	1.73(1.55,1.93)	1.59(1.41,1.81)	1.49(1.34,1.66)	1.36(1.21,1.52)	1.00	<0.001
	Multivariate Adjusted RR (95% CI)	1.95(1.72,2.20)	1.69(1.51,1.89)	1.57(1.39,1.79)	1.48(1.33,1.65)	1.36(1.21,1.52)	1.00	<0.001
Pleura Cancer								
	Cases, No.	17	40	17	30	16	12	
	Age Adjusted Rate b	12.32	11.64	8.53	6.74	3.57	2.65	
	Age Adjusted RR (95% CI)	4.01(1.91,8.42)	4.41(2.31,8.41)	3.21(1.53,6.73)	2.55(1.31,4.98)	1.35(0.64,2.86)	1.00	<0.001
	Age, Smoking Adjusted RR(95%CI)	4.15(1.97,8.77)	4.52(2.36,8.65)	3.27(1.56,6.88)	2.64(1.34,5.17)	1.38(0.65,2.93)	1.00	<0.001
	Multivariate Adjusted RR (95% CI)	4.56(2.13,9.75)	4.84(2.51,9.32)	3.45(1.64,7.28)	2.76(1.41,5.44)	1.40(0.66,2.96)	1.00	<0.001
Melanomas of the Skin								
	Cases, No.	87	309	196	517	612	655	
	Age Adjusted Rate b	60.39	90.44	98.92	113.66	136.81	140.22	
	Age Adjusted RR (95% CI)	0.43(0.35,0.54)	0.65(0.57,0.74)	0.70(0.60,0.83)	0.81(0.72,0.91)	0.97(0.87,1.09)	1.00	<0.001
	Age, Smoking Adjusted RR(95%CI)	0.47(0.38,0.59)	0.69(0.60,0.79)	0.75(0.63,0.88)	0.86(0.77,0.97)	1.00(0.90,1.12)	1.00	<0.001
	Multivariate Adjusted RR (95% CI)	0.53(0.42,0.66)	0.72(0.63,0.83)	0.77(0.66,0.91)	0.89(0.79,1.00)	1.01(0.91,1.13)	1.00	<0.001
Bladder Cancer								
	Cases, No.	230	476	241	606	538	449	
	Age Adjusted Rate b	159.21	138.98	121.15	134.12	120.33	97.24	
	Age Adjusted RR (95% CI)	1.56(1.33,1.83)	1.43(1.26,1.62)	1.24(1.06,1.45)	1.38(1.22,1.56)	1.23(1.09,1.40)	1.00	<0.001
	Age, Smoking Adjusted RR(95%CI)	1.20(1.02,1.41)	1.17(1.03,1.33)	1.02(0.87,1.20)	1.13(0.99,1.27)	1.10(0.97,1.25)	1.00	0.021
	Multivariate Adjusted RR (95% CI)	1.20(1.02,1.41)	1.15(1.01,1.31)	1.01(0.86,1.18)	1.12(0.99,1.26)	1.10(0.97,1.24)	1.00	0.031
Kidney Cancer								
	Cases, No.	80	180	91	262	207	214	
	Age Adjusted Rate b	56.49	53.19	46.08	57.55	46.28	46.19	
	Age Adjusted RR (95% CI)	1.24(0.96,1.60)	1.16(0.95,1.42)	1.00(0.79,1.28)	1.26(1.05,1.51)	1.01(0.84,1.23)	1.00	0.033
	Age, Smoking Adjusted RR(95%CI)	1.10(0.85,1.43)	1.07(0.88,1.31)	0.93(0.73,1.19)	1.16(0.97,1.40)	0.97(0.80,1.18)	1.00	0.311
	Multivariate Adjusted RR (95% CI)	0.97(0.74,1.26)	0.97(0.79,1.19)	0.87(0.68,1.11)	1.09(0.91,1.31)	0.96(0.79,1.16)	1.00	0.767
Brain Cancer								
	Cases, No.	21	74	42	89	98	94	
	Age Adjusted Rate b	15.82	21.68	21.34	19.49	21.84	20.00	
	Age Adjusted RR (95% CI)	0.75(0.47,1.21)	1.09(0.80,1.48)	1.06(0.74,1.53)	0.97(0.73,1.30)	1.09(0.82,1.45)	1.00	0.488
	Age, Smoking Adjusted RR(95%CI)	0.83(0.52,1.35)	1.17(0.86,1.60)	1.13(0.79,1.64)	1.04(0.78,1.39)	1.13(0.85,1.50)	1.00	0.923
	Multivariate Adjusted RR (95% CI)	0.82(0.51,1.34)	1.15(0.84,1.57)	1.12(0.77,1.62)	1.04(0.77,1.39)	1.14(0.86,1.51)	1.00	0.827
Lymphoma								
	Cases, No.	109	267	173	375	380	422	
	Age Adjusted Rate b	76.63	78.24	87.30	82.58	84.96	90.57	
	Age Adjusted RR (95% CI)	0.83(0.67,1.03)	0.87(0.74,1.01)	0.96(0.81,1.15)	0.91(0.79,1.05)	0.94(0.82,1.08)	1.00	0.04
	Age, Smoking Adjusted RR(95%CI)	0.81(0.66,1.01)	0.86(0.73,1.00)	0.96(0.80,1.14)	0.91(0.79,1.04)	0.93(0.81,1.07)	1.00	0.025
	Multivariate Adjusted RR (95% CI)	0.76(0.61,0.95)	0.82(0.70,0.96)	0.92(0.77,1.10)	0.88(0.76,1.01)	0.93(0.81,1.07)	1.00	0.003
Leukemia								
	Cases, No.	63	135	74	158	183	171	
	Age Adjusted Rate b	41.97	39.37	37.32	34.92	40.90	36.87	
	Age Adjusted RR (95% CI)	1.14(0.85,1.52)	1.07(0.85,1.34)	1.00(0.76,1.32)	0.95(0.76,1.17)	1.11(0.90,1.36)	1.00	0.607
	Age, Smoking Adjusted RR(95%CI)	1.10(0.82,1.48)	1.04(0.83,1.31)	0.98(0.74,1.29)	0.92(0.74,1.14)	1.09(0.88,1.34)	1.00	0.794
	Multivariate Adjusted RR (95% CI)	1.11(0.83,1.50)	1.04(0.83,1.31)	0.97(0.74,1.28)	0.92(0.74,1.14)	1.09(0.88,1.35)	1.00	0.787
Localized Prostate Cancer e								
	Cases, No.	938	2435	1436	3311	3727	3734	
	Age Adjusted Rate b	634.94	711.51	724.16	729.10	834.27	802.77	
	Age Adjusted RR (95% CI)	0.79(0.74,0.85)	0.89(0.84,0.93)	0.90(0.84,0.95)	0.91(0.87,0.95)	1.03(0.99,1.08)	1.00	<0.001
	Age, Smoking Adjusted RR(95%CI)	0.83(0.77,0.89)	0.91(0.87,0.96)	0.92(0.87,0.98)	0.94(0.89,0.98)	1.05(1.00,1.10)	1.00	<0.001
	Multivariate Adjusted RR (95% CI)	0.85(0.79,0.92)	0.94(0.89,0.99)	0.94(0.89,1.00)	0.95(0.90,0.99)	1.06(1.01,1.11)	1.00	<0.001
Advanced Prostate Cancer e								
	Cases, No.	124	297	153	414	460	491	
	Age Adjusted Rate b	91.55	87.17	77.57	90.41	103.01	104.39	
	Age Adjusted RR (95% CI)	0.84(0.69,1.02)	0.83(0.72,0.96)	0.74(0.61,0.88)	0.86(0.76,0.99)	0.98(0.86,1.11)	1.00	0.001
	Age, Smoking Adjusted RR(95%CI)	0.85(0.70,1.04)	0.84(0.73,0.98)	0.74(0.62,0.89)	0.88(0.77,1.00)	0.99(0.87,1.12)	1.00	0.002
	Multivariate Adjusted RR (95% CI)	0.89(0.72,1.09)	0.87(0.75,1.01)	0.76(0.63,0.91)	0.89(0.78,1.01)	1.00(0.88,1.13)	1.00	0.013

Abbreviations: RR, relative risk; CI, confidence interval; BMI, body mass index (calculated as weight kilograms divided by height in meters squared).

a p for trend across education groups.

b Age-adjusted Incidence rates are per 100,000 person-years by 5 year age intervals.

c Multivariate models included the following covariates: age (yrs); race (White, Black, Hispanic and Asian, Pacific Islanders and Native Americans, combined); smoking (Never, Quit < = 1 pack per day, Quit>1 pack per day, Currently smoking < = 1 pack per day, Currently Smoking>1 pack per day); alcohol consumptions g/day (0; 0.1−<5, 5−<15, 15<30, 30+); energy (Kcal/day);BMI (<25, 25−<30, 30−<35, 35+); Physical activity (Frequency of at least 20 minutes that caused increases in breathing or heart rate, or worked up a sweat: Never/Rarely, 1–3 time per month, 1–2 times per week, 3–4 times per week, 5+ times per week, Unknown), married (yes/no); family history of cancer (yes/no).

d Smoking related cancers include sites: head neck, esophageal, lung, pancreas, bladder.

e For the sites of colon and prostate (local and advanced) models were adjusted for screening behavior.

Among women, the age–adjusted risk of any cancer for participants with less than high school compared to those with postgraduate education was reduced (0.93, 0.87–0.99) (Table 3). For smoking–related cancers combined, however, the age–adjusted risk for less than high school vs. postgraduate education was increased (1.66, 1.43–1.92) (Table 3). With regard to site-specific malignancies, less educated women had higher risks of colon (1.60, 1.24–2.5), lung (2.14,1.79–2.56), and kidney cancers (1.68, 1.12–2.54), whereas they were at lower risk of developing invasive breast (0.72, 0.61–0.84) and endometrial (0.67, 0.51–0.89) tumors, as well as melanoma of the skin (0.33, 0.22–0.51) (Table 3).

**Table 3:** Relative Risk (RR) of Total Incident Cancer and of Site-Specific Cancer by Educational Attainment in Women.

Educational Attainment								p-trend a
		Less than High School	Completed High School	Post High School Training	Some College	College Graduate	Post Graduate	
All Cancer								
	Cases, No.	1228	4837	1983	4676	2781	2862	
	Age Adjusted Rate b	1321.06	1300.76	1311.88	1358.01	1378.40	1394.23	
	Age Adjusted RR(95%CI)	0.93(0.87,0.99)	0.93(0.89,0.97)	0.94(0.89,0.99)	0.97(0.93,1.02)	0.99(0.94,1.04)	1.00	0.001
	Age, Smoking Adjusted RR(95%CI)	0.86(0.80,0.92)	0.89(0.85,0.93)	0.89(0.84,0.94)	0.91(0.87,0.96)	0.96(0.91,1.02)	1.00	<0.001
	Multivariate Adjusted RR(95%CI) c	0.84(0.79,0.90)	0.88(0.84,0.92)	0.88(0.83,0.93)	0.91(0.87,0.95)	0.96(0.92,1.02)	1.00	<0.001
Smoking Related Cancer d								
	Cases, No.	326	1017	401	947	452	403	
	Age Adjusted Rate b	353.27	270.30	264.98	276.22	229.34	201.12	
	Age Adjusted RR(95%CI)	1.66(1.43,1.92)	1.35(1.20,1.51)	1.32(1.15,1.51)	1.38(1.23,1.55)	1.13(0.99,1.30)	1.00	<0.001
	Age, Smoking Adjusted RR(95%CI)	1.18(1.02,1.37)	1.10(0.98,1.23)	1.03(0.90,1.19)	1.07(0.95,1.20)	1.02(0.89,1.17)	1.00	0.013
	Multivariate Adjusted RR(95%CI)	1.19(1.02,1.38)	1.10(0.98,1.24)	1.05(0.91,1.20)	1.07(0.96,1.21)	1.02(0.89,1.17)	1.00	0.012
Head and Neck Cancer								
	Cases, No.	22	83	35	71	35	30	
	Age Adjusted Rate b	26.47	22.14	23.14	20.55	17.31	13.87	
	Age Adjusted RR(95%CI)	1.61(0.92,2.79)	1.54(1.01,2.34)	1.59(0.97,2.59)	1.41(0.92,2.16)	1.18(0.73,1.93)	1.00	0.024
	Age, Smoking Adjusted RR(95%CI)	1.08(0.62,1.87)	1.21(0.79,1.83)	1.20(0.74,1.96)	1.07(0.70,1.64)	1.05(0.65,1.71)	1.00	0.505
	Multivariate Adjusted RR(95%CI)	1.21(0.69,2.13)	1.33(0.87,2.03)	1.33(0.81,2.17)	1.13(0.74,1.73)	1.07(0.66,1.74)	1.00	0.22
Stomach Cancer								
	Cases, No.	12	48	16	33	15	20	
	Age Adjusted Rate b	10.95	12.67	10.58	9.73	7.40	10.71	
	Age Adjusted RR(95%CI)	1.13(0.55,2.31)	1.21(0.72,2.04)	1.02(0.53,1.97)	0.95(0.54,1.65)	0.76(0.39,1.48)	1.00	0.281
	Age, Smoking Adjusted RR(95%CI)	1.05(0.51,2.16)	1.18(0.70,1.99)	1.00(0.52,1.94)	0.91(0.52,1.58)	0.75(0.38,1.46)	1.00	0.372
	Multivariate Adjusted RR(95%CI)	0.92(0.44,1.92)	1.14(0.67,1.94)	1.00(0.52,1.94)	0.90(0.52,1.58)	0.75(0.39,1.47)	1.00	0.622
Colon Cancer e								
	Cases, No.	113	363	164	325	178	139	
	Age Adjusted Rate b	114.73	95.73	108.15	95.68	90.69	71.20	
	Age Adjusted RR(95%CI)	1.60(1.24,2.05)	1.35(1.11,1.65)	1.54(1.22,1.93)	1.36(1.11,1.66)	1.29(1.04,1.61)	1.00	<0.001
	Age, Smoking Adjusted RR(95%CI)	1.56(1.21,2.00)	1.34(1.10,1.63)	1.50(1.20,1.89)	1.32(1.08,1.61)	1.28(1.03,1.60)	1.00	0.001
	Multivariate Adjusted RR(95%CI)	1.37(1.06,1.77)	1.24(1.01,1.51)	1.44(1.14,1.80)	1.28(1.05,1.57)	1.26(1.01,1.58)	1.00	0.029
Rectum Cancer								
	Cases, No.	34	127	56	116	67	61	
	Age Adjusted Rate b	34.84	33.81	37.06	33.67	34.40	30.11	
	Age Adjusted RR(95%CI)	1.16(0.76,1.77)	1.12(0.82,1.52)	1.22(0.85,1.76)	1.12(0.82,1.53)	1.11(0.79,1.57)	1.00	0.447
	Age, Smoking Adjusted RR(95%CI)	1.09(0.72,1.67)	1.09(0.80,1.48)	1.18(0.82,1.71)	1.08(0.79,1.48)	1.10(0.78,1.56)	1.00	0.657
	Multivariate Adjusted RR(95%CI)	1.05(0.68,1.62)	1.06(0.78,1.45)	1.17(0.81,1.69)	1.08(0.79,1.47)	1.10(0.77,1.55)	1.00	0.822
Pancreatic Cancer								
	Cases, No.	26	97	44	100	52	61	
	Age Adjusted Rate b	26.15	25.31	29.01	29.34	26.73	29.85	
	Age Adjusted RR(95%CI)	0.84(0.53,1.34)	0.83(0.60,1.14)	0.94(0.64,1.39)	0.95(0.69,1.31)	0.86(0.60,1.25)	1.00	0.375
	Age, Smoking Adjusted RR(95%CI)	0.76(0.48,1.20)	0.78(0.57,1.08)	0.88(0.60,1.30)	0.88(0.64,1.22)	0.84(0.58,1.21)	1.00	0.168
	Multivariate Adjusted RR(95%CI)	0.74(0.47,1.19)	0.79(0.57,1.09)	0.88(0.60,1.30)	0.89(0.64,1.22)	0.84(0.58,1.21)	1.00	0.171
Lung Cancer								
	Cases, No.	245	687	265	651	298	237	
	Age Adjusted Rate b	262.84	183.39	175.24	189.69	151.23	119.69	
	Age Adjusted RR(95%CI)	2.14(1.79,2.56)	1.55(1.34,1.80)	1.49(1.25,1.77)	1.62(1.39,1.87)	1.27(1.07,1.51)	1.00	<0.001
	Age, Smoking Adjusted RR(95%CI)	1.43(1.20,1.71)	1.22(1.05,1.41)	1.11(0.93,1.32)	1.19(1.03,1.38)	1.12(0.94,1.33)	1.00	<0.001
	Multivariate Adjusted RR(95%CI)	1.43(1.19,1.72)	1.22(1.05,1.42)	1.12(0.94,1.33)	1.20(1.03,1.39)	1.12(0.94,1.33)	1.00	<0.001
Melanomas of the Skin								
	Cases, No.	25	201	79	200	150	168	
	Age Adjusted Rate b	26.13	54.02	52.23	57.91	73.65	81.66	
	Age Adjusted RR(95%CI)	0.33(0.22,0.51)	0.67(0.55,0.82)	0.64(0.49,0.84)	0.71(0.58,0.88)	0.91(0.73,1.13)	1.00	<0.001
	Age, Smoking Adjusted RR(95%CI)	0.34(0.23,0.53)	0.69(0.56,0.85)	0.66(0.50,0.86)	0.73(0.59,0.89)	0.92(0.73,1.14)	1.00	<0.001
	Multivariate Adjusted RR(95%CI)	0.40(0.26,0.62)	0.71(0.57,0.87)	0.67(0.51,0.88)	0.74(0.60,0.91)	0.93(0.74,1.15)	1.00	<0.001
Bladder Cancer								
	Cases, No.	26	130	47	110	55	60	
	Age Adjusted Rate b	27.64	34.25	30.97	32.28	27.91	30.08	
	Age Adjusted RR(95%CI)	0.85(0.54,1.35)	1.12(0.83,1.53)	1.02(0.70,1.49)	1.07(0.78,1.46)	0.93(0.64,1.33)	1.00	0.886
	Age, Smoking Adjusted RR(95%CI)	0.69(0.43,1.10)	1(0.73,1.36)	0.87(0.59,1.28)	0.90(0.65,1.23)	0.86(0.60,1.24)	1.00	0.404
	Multivariate Adjusted RR(95%CI)	0.67(0.42,1.07)	0.98(0.72,1.34)	0.86(0.59,1.27)	0.89(0.65,1.23)	0.87(0.60,1.25)	1.00	0.306
Kidney Cancer								
	Cases, No.	41	98	39	93	36	52	
	Age Adjusted Rate b	41.70	26.22	25.95	27.20	17.41	25.78	
	Age Adjusted RR(95%CI)	1.68(1.12,2.54)	1.03(0.73,1.44)	1.01(0.67,1.53)	1.06(0.75,1.49)	0.70(0.46,1.07)	1.00	0.006
	Age, Smoking Adjusted RR(95%CI)	1.61(1.07,2.44)	1.01(0.72,1.42)	0.98(0.65,1.49)	1.02(0.72,1.43)	0.69(0.45,1.06)	1.00	0.011
	Multivariate Adjusted RR(95%CI)	1.29(0.85,1.98)	0.89(0.63,1.25)	0.87(0.58,1.33)	0.95(0.67,1.33)	0.69(0.45,1.06)	1.00	0.179
Thyroid Cancer								
	Cases, No.	11	62	15	50	30	25	
	Age Adjusted Rate b	11.86	16.81	9.80	14.38	14.43	11.87	
	Age Adjusted RR(95%CI)	1.03(0.51,2.10)	1.44(0.90,2.29)	0.84(0.44,1.59)	1.21(0.75,1.96)	1.22(0.72,2.08)	1.00	0.542
	Age, Smoking Adjusted RR(95%CI)	1.07(0.52,2.18)	1.50(0.94,2.39)	0.87(0.46,1.66)	1.24(0.77,2.01)	1.24(0.73,2.11)	1.00	0.435
	Multivariate Adjusted RR(95%CI)	1.01(0.49,2.08)	1.45(0.90,2.33)	0.86(0.45,1.63)	1.23(0.76,1.99)	1.26(0.74,2.14)	1.00	0.609
Lymphoma								
	Cases, No.	64	236	87	213	121	128	
	Age Adjusted Rate b	71.15	61.72	57.25	62.25	61.34	62.77	
	Age Adjusted RR(95%CI)	1.04(0.77,1.40)	0.99(0.80,1.23)	0.91(0.69,1.19)	0.98(0.79,1.22)	0.96(0.75,1.23)	1.00	0.855
	Age, Smoking Adjusted RR(95%CI)	1.01(0.75,1.37)	0.98(0.79,1.21)	0.90(0.68,1.18)	0.97(0.78,1.21)	0.95(0.74,1.22)	1.00	0.988
	Multivariate Adjusted RR(95%CI)	0.99(0.72,1.34)	0.94(0.76,1.18)	0.88(0.67,1.15)	0.96(0.77,1.20)	0.95(0.74,1.22)	1.00	0.772
Leukemia								
	Cases, No.	24	77	38	68	31	40	
	Age Adjusted Rate b	22.06	20.60	25.12	19.73	15.66	19.75	0.162
	Age Adjusted RR(95%CI)	1.27(0.76,2.11)	1.04(0.71,1.53)	1.27(0.82,1.99)	1.01(0.68,1.49)	0.79(0.49,1.26)	1.00	0.169
	Age, Smoking Adjusted RR(95%CI)	1.27(0.76,2.11)	1.04(0.71,1.53)	1.26(0.81,1.97)	1.00(0.67,1.47)	0.78(0.49,1.25)	1.00	0.401
	Multivariate Adjusted RR(95%CI)	1.14(0.68,1.93)	0.97(0.66,1.43)	1.19(0.76,1.85)	0.96(0.65,1.42)	0.78(0.49,1.26)	1.00	
Invasive Breast Cancer e								
	Cases, No.	202	876	372	924	574	626	
	Age Adjusted Rate b	332.68	383.29	400.06	434.73	462.79	482.76	
	Age Adjusted RR(95%CI)	0.72(0.61,0.84)	0.79(0.71,0.87)	0.81(0.72,0.93)	0.88(0.80,0.98)	0.93(0.83,1.04)	1.00	<0.001
	Age, Smoking Adjusted RR(95%CI)	0.70(0.60,0.82)	0.78(0.70,0.86)	0.80(0.71,0.91)	0.87(0.78,0.96)	0.93(0.83,1.04)	1.00	<0.001
	Multivariate Adjusted RR(95%CI)	0.83(0.70,0.98)	0.87(0.78,0.97)	0.88(0.77,1.00)	0.93(0.84,1.04)	0.98(0.87,1.10)	1.00	0.002
Cervical Cancer								
	Cases, No.	11	32	11	30	13	15	
	Age Adjusted Rate b	13.96	8.91	7.36	8.51	6.51	7.19	
	Age Adjusted RR(95%CI)	1.85(0.84,4.04)	1.29(0.70,2.40)	1.05(0.48,2.30)	1.23(0.66,2.29)	0.89(0.42,1.87)	1.00	0.083
	Age, Smoking Adjusted RR(95%CI)	1.63(0.74,3.57)	1.19(0.64,2.21)	0.95(0.44,2.08)	1.12(0.60,2.09)	0.85(0.41,1.79)	1.00	0.163
	Multivariate Adjusted RR(95%CI)	1.20(0.52,2.74)	0.95(0.49,1.83)	0.86(0.39,1.93)	1.04(0.54,1.98)	0.82(0.38,1.75)	1.00	0.646
Endometrial Cancer f								
	Cases, No.	65	307	117	285	194	215	
	Age Adjusted Rate b	72.72	82.71	77.52	82.55	94.16	103.78	
	Age Adjusted RR(95%CI)	0.67(0.51,0.89)	0.80(0.67,0.96)	0.75(0.60,0.93)	0.79(0.66,0.95)	0.92(0.76,1.11)	1.00	0.001
	Age, Smoking Adjusted RR(95%CI)	0.72(0.54,0.95)	0.83(0.69,0.98)	0.77(0.62,0.97)	0.82(0.69,0.98)	0.93(0.77,1.13)	1.00	0.003
	Multivariate Adjusted RR(95%CI)	0.65(0.49,0.87)	0.77(0.65,0.93)	0.75(0.60,0.95)	0.85(0.71,1.02)	0.99(0.82,1.21)	1.00	<0.001
Ovarian Cancer e ^,^ g								
	Cases, No.	32	123	53	125	98	73	
	Age Adjusted Rate b	37.19	32.72	35.14	36.07	48.77	35.08	
	Age Adjusted RR(95%CI)	0.96(0.64,1.46)	0.94(0.70,1.25)	0.99(0.69,1.41)	1.02(0.77,1.36)	1.36(1.01,1.85)	1.00	0.175
	Age, Smoking Adjusted RR(95%CI)	0.98(0.65,1.49)	0.94(0.70,1.26)	1.00(0.70,1.42)	1.03(0.77,1.38)	1.37(1.01,1.86)	1.00	0.202
	Multivariate Adjusted RR(95%CI)	1.08(0.70,1.67)	1.00(0.74,1.36)	1.07(0.75,1.54)	1.11(0.83,1.49)	1.43(1.05,1.94)	1.00	0.476

Abbreviations: RR, relative risk; CI, confidence interval; BMI, body mass index (calculated as weight kilograms divided by height in meters squared); MHT, menopausal hormone therapy.

a p for trend across education groups.

b Age-adjusted Incidence rates are per 100,000 person-years by 5 year age intervals.

c Multivariate models included the following covariates: age (yrs); race (White, Black, Hispanic and Asian, Pacific Islanders and Native Americans, combined); smoking (Never, Quit < = 1 pack per day, Quit>1 pack per day, Currently smoking < = 1 pack per day, Currently Smoking>1 pack per day); alcohol consumptions g/day (0; 0.1−<5, 5 −<15, 15<30, 30+); energy (Kcal/day);BMI (<25, 25−<30, 30−<35, 35+); Physical activity (Frequency of at least 20 minutes that caused increases in breathing or heart rate, or worked up a sweat: Never/Rarely, 1–3 time per month, 1–2 times per week, 3–4 times per week, 5+ times per week, Unknown), married (yes/no); family history of cancer (yes/no); MHT use (Current, Former, Never). In female specific anatomical sites, a combined variable of age at first birth and number of children was included.

d Smoking related cancers include sites: head neck, esophageal, lung, pancreas, bladder.

e For the sites of colon, breast and ovarian models were adjusted for screening behavior.

f Women who reported a hysterectomy were excluded from the statistical models for endometrial cancer.

g Women with both ovaries removed were excluded from the statistical models for ovarian cancer.

### Adjustment for smoking and other risk factors

Compared to the age–adjusted results, site specific risk estimates from models that were further adjusted for smoking habits were somewhat attenuated, but remained statistically significant (Tables 2 and 3). Following adjustment for all factors, we found that the education-cancer associations were further attenuated but remained inverse and statistically significant for a number of malignant outcomes, especially for smoking–related cancers combined in men (1.54, 1.42–1.68) and women (1.19, 1.02–1.38 (Tables 2 and 3). Furthermore, positive education associations persisted for localized prostate cancer in men and invasive breast and endometrial cancers in women (Tables 2 and 3).

Among rarer cancers, pleural tumors were strongly and inversely associated with education in men (multivariate model: 4.56. 2.13–9.75).

The smoking-related cancer data suggested effect modification for smoking status itself: cross-product terms for education and smoking were statistically significant for both men (p<0.0001) and women (p = 0.0019). Stratified analyses showed that the inverse association between education and smoking-related cancers association was not present among never smokers but was restricted to current and former smokers. No effect modification was apparent for age, race, body mass index, physical activity, alcohol consumption, birth cohort (50–59 vs. 60+), self reported health (excellent, very good or good vs. fair or poor) and preexisting disease (yes vs. no) (data not shown).”

## Discussion

In this large prospective cohort of United States men and women aged 50 to 71, substantial inverse education gradients persist for incident cancer. In fully adjusted models, we found higher risks among the least, compared to the most, educated individuals, especially for combined smoking–related cancers (comprising those of the head and neck, esophagus, lung, bladder, and pancreas). In addition, we found inverse education gradients for cancers of the stomach and rectum (men only) and colon (women only). Some direct associations with education and cancer risk also emerged, notably those for melanoma of the skin (both men and women), localized prostate cancer (men), and invasive breast and endometrial cancer (women).

The NIH-AARP cohort is a large prospective cohort with detailed information on a variety of covariates which allowed us to control for multiple risk factors at the individual level in an analysis of first primary rare and common malignancies in both men and women. Other prospective studies conducted in Europe have reported similar results, although these studies did not control for risk factors[3], analyzed only common cancers[11], or presented data only for women.[4] Other studies in the U.S. have reported on the relation of education to cancer mortality, with results broadly similar to ours.[23] The availability of registry–based incidence data in our cohort focused the analysis on potential cancer causation, largely circumventing the complicating influence of treatment factors on cancer mortality outcomes.

The smoking–adjusted analyses are revealing in two ways. First, for some sites, particularly lung and smoking–related cancers combined, adjustment for smoking leads to substantial attenuation of the inverse education–cancer association in men and women. Given that smoking is clearly related to education (Table 1) and smoking is an established cause of these cancers, this relative risk attenuation suggests strongly that smoking is a key intermediate factor on the education–cancer pathway. Second, although the education–cancer relative risks are attenuated by smoking adjustment, they do not revert to the null. Even after adjustment for smoking, the lung, esophageal and overall smoking–related cancer risks for the least, compared to the most, educated men remain approximately doubled. This may reflect residual confounding by smoking or the presence of causal factors other than smoking (be they biological or psycho–social) on the education–cancer pathway. That education, even after taking smoking and other factors into account, should consistently predict, for example, the development of esophageal cancer in men remains both tantalizing and a target for etiologic research.

After adjustment for age and smoking, the inclusion of other covariates in the regression models resulted in little additional attenuation of the education–cancer associations. Although residual confounding for such imperfectly measured variables as total energy intake, alcohol consumption, and physical activity cannot be ruled out, these additional factors explain relatively little of the education–cancer connection.

We did not have information on H. pylori infection status to incorporate in the multivariate analyses of gastric cancer. However, when investigated by Nagel et al in a large nested case control study in Europe, the inverse association of gastric cancer remained, albeit non-significant, even after controlling for H. pylori[19].

The data reveal a strong inverse gradient for pleural cancer in men. This finding from a prospective cohort study, possible only because of the study's large size, appears unexplained by smoking and may reflect occupational or environmental exposure to asbestos.[20] It is noteworthy that asbestos was used widely in the United States until the implementation of the Occupational Safety and Health Administration (OSHA) regulations in 1971, when the study participants were approximately aged 26–47 and thus of sufficient age to have accrued occupational or environmental exposure.

Education level was weakly but significantly positively associated with invasive breast cancers in women, which is consistent with findings from other studies.[6], [21] Age at first birth, parity, and use of MHT are all related both to education and breast cancer, which likely accounts for the modest attenuation of the positive education–breast cancer relation in the multivariate analyses. In contrast to some other studies, endometrial cancer was directly related to educational attainment and this association was not attenuated after adjustment for BMI and MHT in the multivariate analyses.[3], [4] The modest overall positive association between all cancer incidence and educational attainment appears to be largely driven by the positive associations for breast and endometrial malignancies.

Studies of educational attainment and prostate cancer have yielded inconsistent results. In our cohort, the education–prostate cancer association was weakly positive, statistically significantly so only for localized disease. The point estimates were similar for localized and advanced prostate cancer, however; the power to detect the positive association with advanced disease was limited. The weak positive association for prostate cancer was largely unaffected by multivariate analysis, which is not surprising given the paucity of strong risk factors for this malignancy.

The direct association of education level with melanoma of the skin in our cohort is in line with previous findings. [22] In general, higher SES individuals are more likely to participate in outdoor leisure activities and vacation in places with high sun exposure[22], and for this reason may have increased melanoma risk.

It is important to note that the AARP membership tends to be more educated, on the average, than the U.S. population as a whole. Nevertheless, the cohort has a wide range of educational attainment, including over 30,000 people, or 6.6% of the study population, with less than a high school education. This wide range of educational attainment allows us to make informative comparisons of cancer incidence across education categories.

Education captures many aspects of the constructs ‘social class’ and ‘socioeconomic status’ and is widely used as an indicator of social ‘difference’ in epidemiologic studies. A particular advantage of investigating education is avoiding reverse causation bias: incident cancer may lead to downward occupational mobility and reduced income but generally will not affect educational status achieved by early adulthood.

In summary, the data from the NIH–AARP cohort show that substantial education gradients in incident cancer risk persist in the United States. A few malignancies are positively associated with educational attainment; these positive associations are primarily of etiologic interest, given that lowering educational attainment is hardly an appropriate strategy for preventing melanoma of the skin or cancers of the breast, prostate, and endometrium. The majority of the observed education associations, however, are inverse, and these are evident especially for smoking– related malignancies. Smoking likely accounts for some—although not all––of the increased cancer risk among lower educated men and women. To the extent that smoking is the mediating causal factor, reducing the differential in smoking rates is a reasonable strategy for addressing SES–cancer inequalities. To the extent that smoking does not account for the inverse associations, further research to identify the causal factors underlying the education–cancer gradients is clearly warranted.

The persistent education-cancer differences in the United States (and many other countries) remain a cause for concern. They also, however, present an opportunity to understand more deeply the etiology of cancer and ultimately reduce its incidence.
